# The role of vasculature and blood circulation in zebrafish swimbladder development

**DOI:** 10.1186/1471-213X-10-3

**Published:** 2010-01-14

**Authors:** Cecilia Lanny Winata, Svetlana Korzh, Igor Kondrychyn, Vladimir Korzh, Zhiyuan Gong

**Affiliations:** 1 Department of Biological Sciences, National University of Singapore, Singapore; 2 Laboratory of Fish Developmental Biology, Cancer and Developmental Cell Biology Division, Institute of Molecular and Cell Biology, Singapore

## Abstract

**Background:**

Recently we have performed a detailed analysis of early development of zebrafish swimbladder, a homologous organ of tetrapod lung; however, the events of swimbladder development are still poorly characterized. Many studies have implicated the role of vascular system in development of many organs in vertebrates. As the swimbladder is lined with an intricate network of blood capillaries, it is of interest to investigate the role of the vascular system during early development of swimbladder.

**Results:**

To investigate the role of endothelial cells (ECs) and blood circulation during development of the swimbladder, phenotypes of swimbladder were analysed at three different stages (~2, 3 and 5 dpf [day postfertilization]) in *cloche *(*clo*) mutant and Tnnt2 morphants, in the background of transgenic lines *Et(krt4:EGFP)*^*sq33-2 *^and *Et(krt4:EGFP)*^*sqet3 *^which express EGFP in the swimbladder epithelium and outer mesothelium respectively. Analyses of the three tissue layers of the swimbladder were performed using molecular markers *hb9*, *fgf10a*, *acta2*, and *anxa5 *to distinguish epithelium, mesenchyme, and outer mesothelium. We showed that the budding stage was independent of ECs and blood flow, while early epithelial growth, mesenchymal organization and its differentiation into smooth muscle, as well as outer mesothelial organization, were dependent on ECs. Blood circulation contributed to later stage of epithelial growth, smooth muscle differentiation, and organization of the outer mesothelium. Inflation of the swimbladder was also affected as a result of absence of ECs and blood flow.

**Conclusion:**

Our data demonstrated that the vascular system, though not essential in swimbladder budding, plays an important role in the development of the swimbladder starting from the early growth stage, including mesenchyme organization and smooth muscle differentiation, and outer mesothelial organization, which in turn may be essential for the function of the swimbladder as reflected in its eventual inflation.

## Background

A functional vasculature is important for the survival of vertebrates, as well as for proper embryonic development [1-6]. However, it is difficult to study this structure in most vertebrate models *in vivo *due to the opacity of the embryo as well as the *in utero *development in mammals. In this respect, the zebrafish model has two major advantages. First, due to the external development of its transparent embryos, it is convenient to observe internal structures and perform analysis of vascular development *in vivo*. Second, the small size of the zebrafish embryo does not require high levels of oxygen uptake and thus passive gas diffusion alone is sufficient for its survival for a quite long time even without a functional circulatory system [1,7,8].

The development of the zebrafish swimbladder has been described recently and its developmental events have been shown to be similar to that of early mammalian lung development [9]. As detected by the expression of *hb9 *[9] and GFP in the gut-GFP transgenic line [10], the swimbladder epithelial bud appears at ~36 hpf as an evagination from the gut at the level of 2^nd ^- 3^rd ^somites on the left side of the embryo. After 48 hpf, the swimbladder enters the growth stage, where it starts to elongate rapidly and incorporate two other tissue layers, mesenchyme and outer mesothelium marked by the expression of *fgf10a*/*acta2 *and *anxa5 *respectively. The end result is a structure consisting of a pneumatic duct and a swimbladder main chamber which is inflated at 5 dpf.

During the growth phase, the early interactions between the three tissue layers of the swimbladder are regulated by the Hedgehog pathway [9]. However developmental events after 72 hpf remain largely unexplored. Therefore, a detailed analysis of late swimbladder development will complement our current understanding of swimbladder development and is highly desirable as the swimbladder is inflated and becomes a fully functional organ during this period.

The vascular anatomy of the zebrafish larvae has been described in great detail [11]. The importance of a functional circulatory system in the proper development of several organs has been highlighted in several studies in the zebrafish, including analyses of vasculogenesis in the liver [1,2,5,6]. Since the swimbladder has been shown to be a vascularised organ, it is the aim of our study to characterize the vasculogenesis of the swimbladder in detail as well as to investigate the role of circulation in its development in order to gain a comprehensive understanding of this process. To investigate the requirement of ECs in the development of swimbladder, *clo*^-/- ^mutants lacking the endocardium and ECs [1,7], were analysed using molecular markers specific for all three swimbladder tissue layers. To further elucidate the role of circulation in the development of swimbladder, we performed morpholino knockdown of Tnnt2, the general phenotype of which has been reported to phenocopy the *silent heart *mutant [1,12-14], where blood flow does not occur due to defective cardiac contractility. We used two enhancer trap transgenic lines, *Et(krt4:EGFP)*^*sq33-2 *^and *Et(krt4:EGFP)*^*sqet3 *^[9], with specific EFGP expression in the inner epithelial and outer mesothelial layers of swimbladder, respectively, to perform functional analysis by generating the *clo*^-/- ^mutant on the background of these lines as well as through Tnnt2 morpholino knockdown. Our results demonstrated the requirement of both endothelia and blood flow in sustaining swimbladder growth.

## Results

### Swimbladder vascular system

In order to characterize the vascular anatomy of the swimbladder *in vivo*, as well as to observe the dynamics of blood circulation in the developing swimbladder, we performed two genetic crosses between transgenic lines; *Et(krt4:EGFP)*^*sq33-2 *^and *Tg(fli1:EGFP)*^*y*1^, were crossed to show blood vessels formation around swimbladder and *Et(krt4:EGFP)*^*sq33-2 *^was crossed with *Tg(gata1:dsRed)sd2 *for observation of erythrocytes circulating in the vessels and to identify the initiation of circulation.

**Figure 1:**
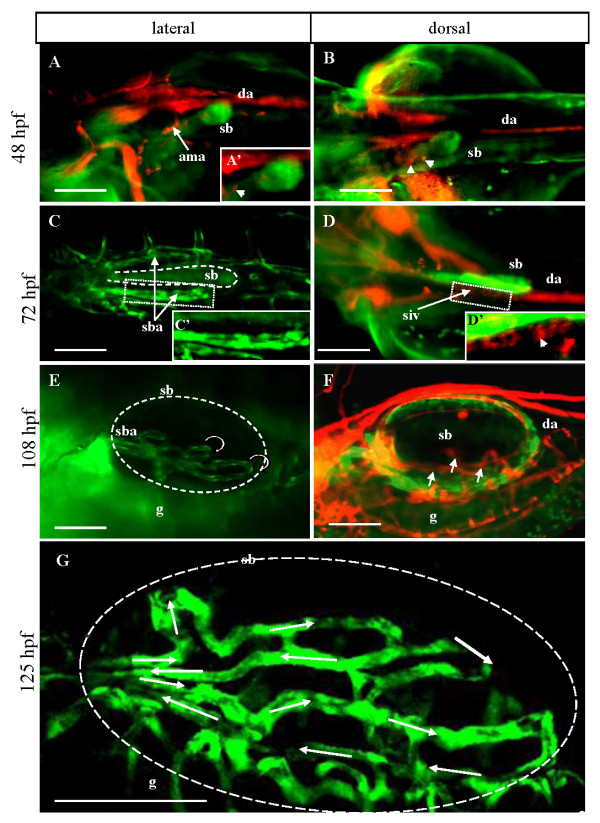
**The vascular anatomy of the larval swimbladder**. (A - B) Erythrocyte-specific RFP expression (white arrowheads) and swimbladder EGFP expression in *Et(krt4:EGFP)*^*sq33-2 *^× *Tg(gata1:dsRed)sd2 *double transgenic larvae at 48 hpf, showing the active circulation in the vicinity of the developing swimbladder bud. (C - D) Blood circulation and swimbladder bud at 72 hpf in *Et(krt4:EGFP)*^*sq33-2 *^× *Tg(fli1:EGFP)*^*y*1 ^(C) and *Et(krt4:EGFP)*^*sq33-2 *^× *Tg(gata1:dsRed)sd2 *(D) double transgenic larvae. A pair of swimbladder artery (sba), as visualized by the GFP expression, could be seen to develop towards the swimbladder bud from both lateral sides in (C). (C', D') 2× magnification of the boxed regions in (C) and (D), respectively. Blood circulation was present in this vessel as observed in *Et(krt4:EGFP)*^*sq33-2 *^× *Tg(gata1:dsRed)sd2 *double transgenic larvae (D). (E - F) Blood vessels (*rete mirabile*) in the swimbladder in *Et(krt4:EGFP)*^*sq33-2 *^× *Tg(fli1:EGFP)*^*y*1 ^(E) and *Et(krt4:EGFP)*^*sq33-2 *^× *Tg(gata1:dsRed)sd2 *(F) double transgenic larvae at 108 hpf. Each swimbladder artery contains two main countercurrent vessels formed through looping at the caudal end of the swimbladder (circular arrows in E). Blood circulation could be observed in the *rete mirabile *in *Et(krt4:EGFP)*^*sq33-2 *^× *Tg(gata1:dsRed)sd2 *double transgenic larvae (white arrows in F). (G) A close-up view of the network of swimbladder vessels at 125 hpf. Direction of blood flow in these vessels is indicated by white arrows. Abbreviations: ama, anterior mesenteric artery; da, dorsal aorta; g, gut; sb, swimbladder; sba, swimbladder artery; siv, supra-intestinal vessel. Scale bars: 250 μm in (A-F), 100 μm in (G).

Our observations of 48 hpf embryos revealed that the swimbladder bud was not in contact with blood vessels (Figure 1A and 1B). Circulation in the nearby anterior mesenteric artery, which branched off into smaller supra-intestinal vessels, could be observed just anterior and ventral to the swimbladder (Figure 1A). By 3 dpf, a large vessel with active circulation started to grow towards the swimbladder, although the smaller capillaries in contact with the swimbladder had not appeared yet (Figure 1C and 1D). At the same time, the supra intestinal vessels formed more loops ventral to the swimbladder (siv in Figure 1C, 1C', 1D and 1D'). By 108 hpf, blood circulation through the swimbladder capillaries penetrated the fully developed swimbladder (sba in Figure 1E, white arrows in Figure 1F). Upon initial inflation of the swimbladder during this period, loops of blood vessels were observed covering its lateral surface (Figure 1E). By 5 dpf, additional loops of vessels could be seen (Figure 1G). These vessels were arranged in countercurrent pairs as a result of looping at their caudal ends (Figure 1E).

### Epithelial budding and early growth is independent of ECs and blood circulation

**Figure 2:**
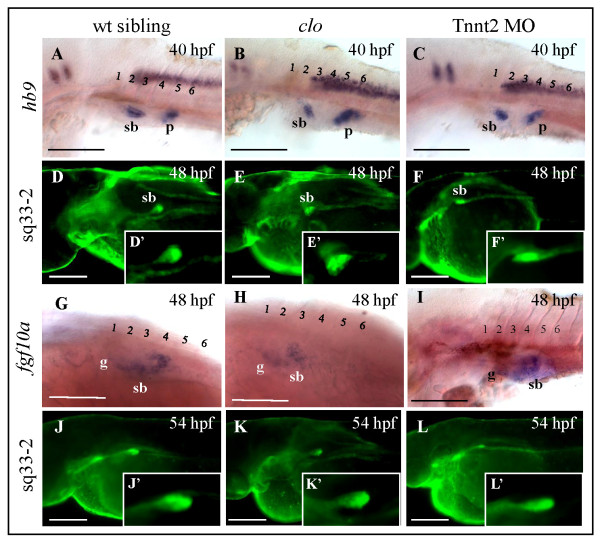
**Role of endothelial cells during swimbladder budding and early growth phase**. (A - C) Expression of swimbladder epithelial marker *hb9 *in control, *clo*^-/- ^mutant, and Tnnt2 morphant at the beginning of budding stage (40 hpf). Note the similar size of epithelial bud in all these embryos. (D - F) EGFP expression in control, *clo*^-/-^, and Tnnt2 morphants on *Et(krt4:EGFP)*^*sq33-2 *^background at 48 hpf. (D' - F') A 2.5× magnification of the swimbladder in (D - F). (G - I) Expression of *fgf10a *mesenchymal marker in swimbladders *clo*^-/-^, mutant, and Tnnt2 morphant embryos was initiated at the same time as that in wild type, at 48 hpf. (J - L) EGFP expression in the same transgenic line during early growth stage at 54 hpf. Note the presence of swimbladder bud which has increased in size in all three larvae observed. (J' - L') A 2.5× magnification of the swimbladder in (J - L). Numbers represent anterior somites, Abbreviations: sb, swimbladder; g, gut; p, pancreas. Scale bars, 250 μm.

Previously, it has been shown that the early swimbladder formation consisted of three phases: budding (36 - 48 hpf), growth (48 hpf - 4.5 dpf) and inflation (4.5 dpf onwards) [9]. In order to investigate whether swimbladder epithelial budding is dependent on ECs, we performed whole mount in situ hybridization analysis using the swimbladder epithelial marker *hb9 *in *clo*^-/- ^mutant and Tnnt2 morphant larvae. At 48 hpf, the body size of *clo*^-/- ^mutant and Tnnt2 morphant embryos are comparable to that of control. Analysis of swimbladder epithelium using *hb9 *expression at this time indicated that budding was not affected by the absence of either ECs or blood circulation as swimbladder epithelium was present in both *clo*^-/- ^mutant and Tnnt2 morphant larvae (Figure 2A - 2C). This observation was also confirmed in *Et(krt4:EGFP)*^*sq33-2 *^transgenic line (Figure 2D - 2F, and 2D' - 2F'). Moreover, we found no significant difference in the size of epithelium in all observed larvae as compared to control (data not shown). The mesenchymal layer was also present as shown by *fgf10a *expression in *clo*^-/- ^mutant and Tnnt2 morphant larvae from 48 hpf, but its organization appeared to be affected in both the mutant and morphant (Figure 2G - 2I). Therefore, by the end of the budding phase, the lack of ECs in *clo*^-/- ^mutants, as well as the absence of blood circulation in Tnnt2 morphants, did not affect the swimbladder epithelium. Mesenchymal specification occurred on time in all observed larvae, though its organization appeared to be dependent on ECs as observed in *clo*^-/- ^mutants.

By 54 hpf, the swimbladder epithelium in all observed larvae increased in size due to cell proliferation with an increased number of cells [9], suggesting that the beginning of growth and initial extension of the swimbladder were similar in these mutant, morphant and wild type control fry (Figure 2J - 2L and 2J' - 2L').

### Contribution of ECs and blood flow to swimbladder development during later growth phase

**Figure 3:**
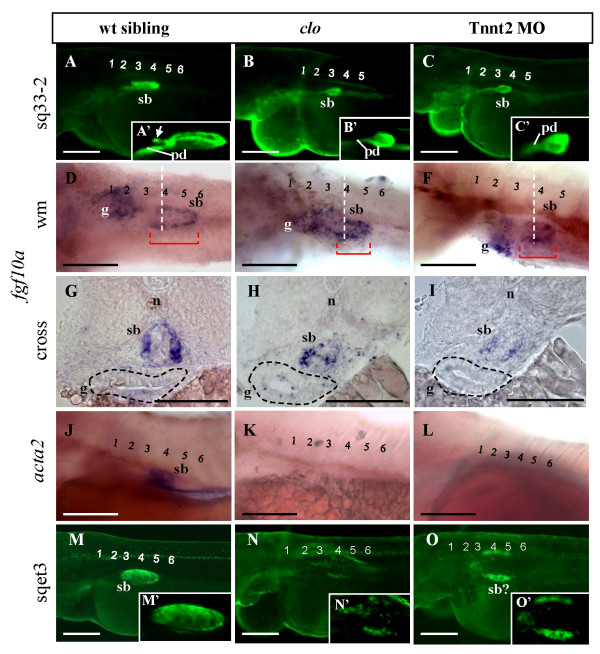
**The role of ECs and blood circulation on swimbladder growth**. (A - C) EGFP expression in control, *clo*^-/-^, mutant, and Tnnt2 morphants on the background of *Et(krt4:EGFP)*^*sq33-2 *^at 72 hpf. Swimbladder appeared smaller in larvae without endocardium and endothelium (*clo*^-/-^, B) and without cardiac function (Tnnt2 MO, C) as compared to wild type (A). (A' - C') 2.5× magnification of the swimbladder in (A - C) respectively. The white arrow indicates the primordium of swimbladder anterior chamber. (D - F) Presence of swimbladder mesenchyme at 72 hpf as detected by *fgf10a *expression in wild type, *clo*^-/- ^mutant, and Tnnt2 morphant. (G - I) Cross section at the levels indicated by white dashed lines in panel (D - F) to show mesenchymal *fgf10a *expression in wild type, *clo*^-/- ^mutant, and Tnnt2 morphant larvae at 72 hpf. Red bar indicates swimbladder length, Gut (g) is demarcated by black dashed line. (J - L) Analysis of *acta2 *expression in swimbladder in wild type (J), *clo*^-/- ^mutant (K) and Tnnt2 morphant (L) at 72 hpf. (M - O) EGFP expression in swimbladder outer mesothelium in wild type (M), *clo*^-/- ^mutant (N), and Tnnt2 morphant (O) at 72 hpf. Note the severe reduction of outer mesothelium in *clo*^-/- ^mutant and its disorganization into two lateral stripes in Tnnt2 morphant. (M' - O') 2.5× magnification of the swimbladder in (M - O) respectively. Numbers indicate anterior somites. Abbreviations: pd, pneumatic duct; n, notochord; sb, swimbladder. Scale bars: 250 μm.

By 72 hpf, the swimbladder epithelium in *clo*^-/-^mutant appeared to be larger than that at 48 hpf, indicating that growth could still occur. However, it did not appear to grow much further after budding, as evident by the presence of a short pneumatic duct, and its small size compared to that of wild type, which more resembled that in 50 - 54 hpf control larvae (Figure 3A and 3B). While the anterior chamber primordium could be observed in wild type, it was absent in *clo*^-/- ^mutants (Figure 3A' and 3B'). These observations suggest that ECs are required during the early stage of swimbladder growth between 54 hpf to 72 hpf. However, the epithelium in Tnnt2 morphant appeared to grow past the budding stage (Figure 3A and 3C), and it possessed a long and apparent pneumatic duct. Nevertheless, the swimbladder epithelium in Tnnt2 morphants did not appear to have the anterior chamber primordium (Figure 3A' and 3C').

Whole mount observations revealed that, at 72 hpf, the mesenchyme in *clo*^-/- ^mutant and Tnnt2 morphant larvae was shorter than that in wild type control larvae (Figure 3D - 3F). Moreover, its organization was interrupted in Tnnt2 morphant larvae and even more so in *clo*^-/- ^mutant (Figure 3G - 3I). Additionally, mesenchymal differentiation into smooth muscle was affected in both *clo*^-/- ^and Tnnt2 morphant larvae, as indicated by the absence of *acta2 *expression (Figure 3J - 3L).

The outer mesothelium in *clo*^-/- ^mutant and Tnnt2 morphant appeared to be disorganized as well (Figure 3M - 3O and 3M' - 3O'), although the two inner layers were present (Figure 3A - 3I). This was also confirmed by the expression of outer mesothelial marker *anxa5 *(data not shown). By 72 hpf, cells positive for EGFP in the *Et(krt4:EGFP)*^*sqet3 *^on the *clo*^-/- ^background could be seen as a disorganized mass of cells around the swimbladder domain (Figure 3N and 3N'). In Tnnt2 morphant, more outer mesothelial cells could be observed; however, they were also disorganized (Figure 3O and 3O').

### Lack of swimbladder inflation in the absence of ECs and blood flow

**Figure 4:**
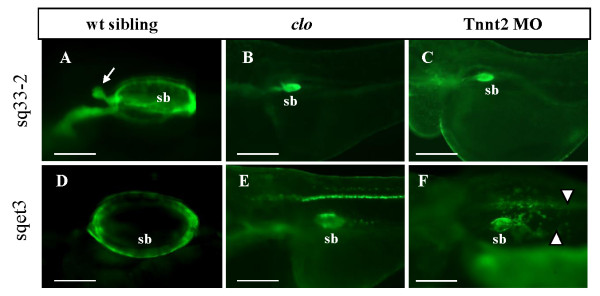
**Swimbladder inflation is affected in the absence of blood circulation**. (A - C) EGFP expression in swimbladder epithelium in control, *clo*^-/- ^mutant, and Tnnt2 morphants on the background of *Et(krt4:EGFP)*^*sq33-2 *^at 5 dpf. (D - F) EGFP expression in swimbladder outer mesothelium in control, *clo*^-/- ^mutant, and Tnnt2 morphant on the background of *Et(krt4:EGFP)*^*sqet3 *^at 5 dpf. All mutants and morphants failed to inflate their swimbladder by 5 dpf. Abbreviation: sb, swimbladder. Scale bars: 250 μm.

At 5 dpf, the swimbladder of *clo*^-/- ^mutant and Tnnt2 morphant larvae remained uninflated (Figure 4A - 4F). Observation in *Et(krt4:EGFP)*^*sq33-2 *^revealed that the swimbladder epithelium in *clo*^-/- ^mutant and Tnnt2 morphant were hypoplastic and only developed until a certain growth stage (Figure 4A - 4C). The outer mesothelium also showed a partially disorganized pattern with scattered cells especially at the posterior part (Figure 4D - 4F).

In summary, ECs and blood circulation are not involved in the specification of the swimbladder epithelium, but are required for its growth at later stages. Although, in the absence of ECs, the mesenchyme develops at the same time as in wild type, it lacks organization and fails to differentiate into smooth muscle. Similarly, these developmental defects do not affect the specification of the outer mesothelium, but they affect the organization of this layer.

## Discussion

### Endothelial cells affect the growth of swimbladder

ECs are main components of the circulatory system. They are specified early during development and subsequently become the building blocks of blood vessels [15-18]. Their involvement in the development of the liver [3,6] and pancreas [4,19] have been extensively described. Our observation of swimbladder in *clo*^-/- ^mutants shows that swimbladder budding is not affected in the absence of ECs, while growth of the swimbladder after bud stage, the organization of the mesenchyme and outer mesothelium, and differentiation of mesenchyme into muscle seem to be dependent on them. The requirement of ECs only for later stages of growth is consistent with that observed in liver development [6]. In our previous study, we have demonstrated that ECs contribute to the formation of sinusoids in liver, which contribute directly to liver size and the organization of hepatocytes [6]. The ability of ECs to induce and regulate the growth of adjacent cells has also been demonstrated in the liver of mice and humans [3,19]. However, although lined with a network of blood capillaries, the swimbladder is structurally very distinct from the liver. First of all, it is a thin uniform layer of cells with a hollow cavity in the center lumen, unlike the liver which is densely packed with hepatocytes. Secondly, ECs interact directly with individual hepatocytes in the liver, forming a specific arrangement of hepatocytes around each sinusoid [6], which does not occur in the swimbladder. Therefore, the contribution of ECs in swimbladder growth is unlikely a structural one.

In the zebrafish, vasculature in the swimbladder is already present by 60 hpf [11]. In *clo*^-/- ^mutants, the swimbladder appeared to be arrested in growth at around 50 - 54 hpf. Thus it seems that during this period, ECs provide certain morphogenic signals which are required for swimbladder growth, and in this way affect the organization of the outer mesothelium. It has been known that ECs could secrete many factors, including mitogenic and chemotactic factors [20-22]. The ability of ECs to induce and regulate growth has also been demonstrated in the liver of mice and humans [3,20]. Therefore, it is possible that these signals may affect cell proliferation and organization of swimbladder and contribute in this manner into its growth.

### A functional blood circulation contributes to normal growth of the swimbladder

A functional blood circulation is known to be essential for liver growth [6]. Therefore it is likely that blood circulation contributes to swimbladder growth as well. Although the exact mechanism is yet to be determined, blood circulation likely stimulates growth through their delivery of nutrients and other factors which induce cell proliferation and possibly differentiation. In agreement with our previous observations on the liver [6], the lack of blood circulation did not affect the budding stage of swimbladder either. Neither did it affect early stages of growth (up to 54 dpf), as the swimbladder bud continued to elongate, forming the pneumatic duct and the main chamber. The systemic blood circulation in zebrafish is initiated at 24 hpf. There has been no investigation into the precise timing of blood flow initiation in the swimbladder vasculature. However, it has been previously observed that the swimbladder artery is already filled with circulating blood by 4 dpf [11]. Our observation of *Tg(gata1:dsRed)*^*sd*2 ^line showed that by 3 dpf, a branch of the anterior mesenteric artery leading to the swimbladder becomes active (Figure 1C). This timing almost coincides with the timing of growth cessation in the swimbladder of Tnnt2 morphants after 54 hpf, as evident by the absence of the anterior chamber primordium in *clo*^-/- ^mutant and Tnnt2 morphant even at 72 hpf; in contrast, the anterior chamber primodium appears at round 65 hpf in wild type fry. This also explains the disorganization of mesenchyme which takes place at around this time, and hence, the disruption observed in the outer mesothelium.

The inability of swimbladder to inflate in Tnnt2 morphants could be a consequence of the lack of blood circulation as well. It is known that the vascular network of the swimbladder carries oxygen and carbon dioxide which could diffuse into the swimbladder. This mechanism serves to maintain the volume of the swimbladder [23-25]. It is possible that the lack of a functional circulation makes it impossible to maintain an inflated state of the swimbladder even after uptake of air bubbles from the surface. However, given the fact that the inflation of the swimbladder depends on surface air-gulping [26] (our unpublished observations), which in turn depends on coordinated activity of several organs, including the nervous and muscular system that could be affected by pan-embryonic knockout of gene activity, it is important to ascertain that the anomaly of swimbladder inflation is linked to changes in its structure. The ability of swimbladder to act as a vessel for gas exchange resembles that of the mammalian lungs. In the lungs, a network of capillaries surrounds the alveoli and oxygen and carbon dioxide could be exchanged between the bloodstream and the atmosphere. Despite the distinct functions of the swimbladder and lungs in aquatic and terrestrial animals, this striking similarity illustrates a possible retention of ancestral features for gas exchange in both organs.

## Conclusions

We have demonstrated that the lack of ECs or blood circulation has no effect on swimbladder development during budding stage, but affects its growth in early growth phase by the lack of ECs and in late growth phase by the lack of blood circulation. The organization of mesenchyme and its subsequent differentiation into smooth muscle, as well as organization of outer mesothelium, were also interrupted by the absence of either ECs or blood circulation. Based on these observations, we conclude that, although not critical for the specification of swimbladder cells, vasculature plays a crucial role in growth of this organ, with ECs contributing to early growth phase and blood circulation to later events of the growth phase. Both ECs and blood circulation are also necessary for the differentiation of mesenchymal smooth muscle and organization of the outer mesothelium in swimbladder, which in turn is essential for the proper functioning of the organ as reflected in its ability to inflate. Thus, our study indicates a similar role of ECs and blood circulation in development of swimbladder and liver, where the absence of ECs and/or blood circulation does not affect budding but growth of these organs [6].

## Methods

### Zebrafish wild type, transgenic, and mutant strains

Wild type zebrafish from AB background, transgenic lines *Et(krt4:EGFP)*^*sq33-2 *^and *Et(krt4:EGFP)*^*sqet3 *^[9], and heterozygous *clo *mutant [1] were maintained in the IMCB zebrafish facility according to the zebrafish book [27] and in compliance with the Institutional Animal Care and Use Committee (IACUC) guidelines. Developmental stages are presented as hours post fertilization (hpf) or days post fertilization (dpf). *Et(krt4:EGFP)*^*sqet3 *^line has been established during the *Tol2 *transposon-mediated enhancer trap screen [28-30]. *Et(krt4:EGFP)*^*sq33-2 *^line was generated after remobilization of the *Tol2 *transposon from the donor site into a new location by injection of transposase mRNA into embryos of transgenic line with a single transposon insertion [31]. Both transgenic lines show GFP expression in the swimbladder, with the EGFP expression in the epithelial layer in *Et(krt4:EGFP)*^*sq33-2 *^and in the outmost mesothelial layer in *Et(krt4:EGFP)*^*sqet3 *^[9].

### Genetic crosses of mutant and transgenic lines

To analyze the requirement of endothelial cells on swimbladder development and growth, we used *cloche *mutants (*clo*^*s*5 ^[point mutation allele]) which lack almost all endothelial cells [1]. Both *Et(krt4:EGFP)*^*sq33-2 *^and *Et(krt4:EGFP)*^*sqet3 *^transgenic lines were crossed with *cloche *heterozygotes to transfer the transgene into the *cloche *mutants. After their progeny reached maturity, these fishes were crossed randomly to identify *cloche *heterozygotes that carry the transgene. These fishes were crossed to obtain homozygous mutants with *Et(krt4:EGFP)*^*sq33-2 *^or *Et(krt4:EGFP)*^*sqet3 *^transgenic background and their development was monitored. In order to visualize the circulatory system in the zebrafish swimbladder, *Et(krt4:EGFP)*^*sq33-2 *^or *Et(krt4:EGFP)*^*sqet3 *^were crossed with *Tg(gata1:dsRed)*^*sd*2 ^transgenic line expressing red fluorescent protein in the red blood cells [32] or *Tg(fli1:EGFP)*^*y*1 ^expressing GFP in the blood vessels [33].

### Whole mount in situ hybridization and histology

Whole mount *in situ *hybridization with Digoxygenin-labeled and Fluorescein-labeled riboprobes and histology was performed using standard protocols as described previously [34].

### Microscopy

To facilitate visualization of swimbladder of larval zebrafish in whole-mount preparations, pigmentation of skin was inhibited with 0.2 mM 1-phenyl-2-thiourea (Sigma, USA) according to established protocols [27]. Observations and photography of live embryos were performed using a dissecting fluorescent microscope (SZX12 Olympus, Japan), a compound microscope (Zeiss Axioscope 2, Zeiss, Germany) and a confocal microscope (Zeiss LSM510, Germany). The two-color images were taken at the same focal plane, using epifluorescence with a Rhodamine filter for the first and FITC filter for the second. These images were then superimposed using Zeiss AxioVision software or Photoshop (Adobe, USA). Three-dimensional confocal projections were generated using Zeiss LSM510 software (Zeiss, Germany). In all confocal studies, at each time point, 5-8 embryos/larvae from random pairs were examined.

### Tnnt2 knockdown

To analyze the role of circulatory defects in swimbladder development, Tnnt2 antisense morpholino oligonucleotide (5'-CATGTTTGCTCTGATCTGACACGCA), which targets the Tnnt2 translation start codon and phenocopies the *silent heart *(*sih) *mutation [15], was obtained from Gene Tools (USA). A total of 4 ng of Tnnt2 morpholino oligonucleotide was injected into 1-2 cell stage of *Et(krt4:EGFP)*^*sq33-2 *^or *Et(krt4:EGFP)*^*sqet3 *^embryos and a stable non-contractile heart phenotype was observed in 98% of injected embryos and larvae from 24 hpf to 7 dpf (the last day of survival). Morphants without obvious motility defect and lacking blood circulation (with pericardial edema) were used for our analysis.

### Histology

For cryosectioning, zebrafish embryos were ice-chilled and fixed with ice-cold 4% paraformaldehyde (PFA) in phosphate-buffered saline (PBS) at 4°C overnight. After fixation, embryos were embedded in 1.5% Bacto agar containing 5% sucrose and incubated in 30% sucrose at 4°C overnight. The embedded embryos were oriented and sectioned with a cryostat microtome (10 μm thickness). For paraffin sections, the embryos and larvae were fixed with either 4% PFA in PBS or Bouin's fixative, followed by paraffin embedding and sectioning (4 μm). Serial sections (from at least 3-5 embryos or larvae at each time point) were de-paraffinized, stained with hematoxylin-eosin, dehydrated, and examined.

## Authors' contributions

CLW - made genetic crosses, systematic analyses of organogenesis and vasculogenesis, and wrote the manuscript, SK - performed morpholino injection, made images, and performed analyses of vasculogenesis in transgenic and mutant fish, IK - generated ET transgenic lines, made confocal images; VK, ZG - developed the concept of the project, wrote and approved the manuscript. All authors read and approved the final manuscript.
